# The correlation between the severity of cerebral microbleeds and serum HMGB1 levels and cognitive impairment in patients with cerebral small vessel disease

**DOI:** 10.3389/fnagi.2023.1221548

**Published:** 2023-06-22

**Authors:** Minghua Wang, Junli Liu, Fan Wang, Qing Li, Jian Zhang, Sibei Ji, Shaomin Li, Chengbiao Lu, Jianhua Zhao

**Affiliations:** ^1^Henan Joint International Research Laboratory of Neurorestoratology for Senile Dementia, Henan Key Laboratory of Neurorestoratology, Neurology, First Affiliated Hospital of Xinxiang Medical University, Xinxiang, China; ^2^Imaging Department, First Affiliated Hospital of Xinxiang Medical University, Xinxiang, China; ^3^Ann Romney Center for Neurologic Diseases, Brigham and Women’s Hospital and Harvard Medical School, Boston, MA, United States; ^4^Sino-UK Joint Laboratory of Brain Function and Injury of Henan Province, Department of Physiology and Neurobiology, Xinxiang Medical University, Xinxiang, China

**Keywords:** cerebral small vessel disease, cerebral microbleeds, HMGB1, cognitive, blood-brain barrier

## Abstract

**Objective:**

The study investigated the correlation and predictive value between the severity of cerebral microbleeds (CMBs) and the level of serum High Mobility Group Protein B1 (HMGB1) and the occurrence of cognitive impairment in patients with cerebral small vessel disease (CSVD).

**Methods:**

A total of 139 patients with CSVD admitted to the Department of Neurology of the First Affiliated Hospital of Xinxiang Medical University from December 2020 to December 2022 were selected as study subjects. The Montreal Cognitive Assessment (MoCA) scale was used to assess the cognitive function and was divided into the cognitive impairment group and the cognitive normal group. Magnetic Resonance Imaging (MRI) and Susceptibility Weighted Imaging (SWI) were used to screen and assess the severity of CMBs. Serum HMGB1 levels of CSVD patients were measured by enzyme linked immunosorbent assay (ELISA). Multivariable logistic regression analysis was used to explore risk factors for cognitive impairment and CMBs. *Pearson* correlation analysis was used to investigate the correlation between HMGB1 and cognitive function. Receiver Operating Characteristics (ROC) curves were used to assess the predictive value of HMGB1 for the occurrence of cognitive impairment in patients with CMBs.

**Results:**

High Mobility Group Protein B1, uric acid (UA), glycosylated hemoglobin (HbA1c), CMBs, lacunar cerebral infarction (LI), years of education, and history of hypertension were risk factors for cognitive impairment (*P* < 0.05); HMGB1 was significantly and negatively associated with total MoCA score, visuospatial/executive ability, and delayed recall ability (*P* < 0.05). HMGB1 was significantly and positively correlated with the number of CMBs (*P* < 0.05). The area under the ROC curve for HMGB1 predicting cognitive impairment in patients with CMBs was 0.807 (*P* < 0.001).

**Conclusion:**

Serum HMGB1 levels are associated with the development of cognitive impairment in CSVD patients, and serum HMGB1 levels have a high predictive value for the development of cognitive impairment in CSVD patients with combined CMBs, which can be used for early clinical identification and intervention of vascular cognitive impairment.

## 1. Introduction

Cerebral small vessel disease refers to a series of clinical, imaging and pathological syndromes caused by various etiologies affecting small arteries and their distal branches, micro-arteries, capillaries, micro-venules and small veins in the brain. CMBs are a type of brain parenchymal damage caused by injury to the micro-vessels of the brain, whose main pathological changes are the deposition of iron-containing hemoglobin at the lesion as well as lipid hyalinosis of the micro-arteries and amyloid deposition in the vessel wall (Ikram et al., 2022). CMBs have been found to be widely present in patients with cognitive impairment due to cerebrovascular disease and neurodegenerative disorders, and there is a correlation between the number of CMBs and their location in the brain and cognitive function. CMBs play an important role in the total load of CSVD imaging markers, are a key factor in the development of cognitive impairment in CSVD patients, and may be involved in the onset and development of cognitive impairment (Wu et al., 2014).

High Mobility Group Protein B1 is an inflammatory factor and damage signaling molecule that affects the structural and functional changes of the blood-brain barrier (BBB) and has a role in regulating innate immunity and inducing the release of other inflammatory factors (Xue et al., 2021). In the presence of central nervous system damage caused by inflammation, neurodegenerative lesions, and vascular damage, HMGB1 is rapidly released and inhibits synaptic plasticity by modulating the inflammatory response, exacerbating the pathological damage to damaged tissues (Ikram et al., 2022), ultimately leading to learning memory decline and cognitive dysfunction.

The objectives of this study were to observe the risk factors for the development of cognitive impairment and CMBs, to investigate the level of HMGBl in patients with CMBs and its correlation with cognitive impairment, to assess the predictive value of HMGB1 for the development of cognitive impairment in patients with CMBs, and to provide valid evidence for the early identification of vascular cognitive impairment due to CSVD.

## 2. Object

A total of 139 patients with CSVD who were hospitalized in the Department of Neurology of the First Affiliated Hospital of Xinxiang Medical University from December 2020 to December 2022 were selected for the study, and the patients were divided into cognitive impairment group (*n* = 72) and cognitive normal group (*n* = 67) according to the cognitive function evaluation results. 61 patients with CMBs were screened according to the results of MRI and SWI tests.

## 3. Methodology

### 3.1. Research subjects

Inclusion criteria:

(1) Age 40∼85 years;
(2) Patients with confirmed diagnosis of CSVD by cranial MRI findings (Shaaban et al., 2019) and must meet the diagnostic criteria for CMBs;
(3) Those who have good cranial MRI and SWI data and laboratory data;
(4) Agree to participate in this study and sign the informed consent form.

Exclusion Criteria:

(1) Atherosclerotic ischemic stroke or cardiogenic embolism in large arteries;
(2) Those with severe organic lesions such as malignant tumors or severe heart, lung, liver and kidney diseases and genetic metabolic disorders.
(3) Those who have mental illness and are on long-term antipsychotic medication.
(4) Patients who refused to cooperate with the study or were unable to sign the informed consent form.

The study was approved by the Ethics Committee of the First Affiliated Hospital of Xinxiang Medical University (approval number: EC-022-078) and registered with the Chinese Clinical Trials Registry (ChiCTR2200060083; 18 May 2022). All study procedures were performed in accordance with the latest version of the Declaration of Helsinki, and all participants signed a written informed consent form.

### 3.2. Clinical data collection

Clinical data were collected from both groups, including gender, age, years of education, medical history (cerebral infarction, cerebral hemorrhage, hypertension, diabetes, heart disease), personal history (smoking, drinking), and peripheral blood biochemical parameters, namely, total cholesterol (TC), triglyceride (TG), high density lipoprotein cholesterol (HDL-C), low density lipoprotein cholesterol (LDL-C), fasting blood glucose (FBG), glycated hemoglobin (HbAlc), homocysteine (Hcy), urea (UREA), creatinine (Cr), uric acid (UA), acrylic aminotransferase (ALT), and aspartate transaminases (AST) levels.

### 3.3. Neuropsychological assessment

The MoCA scale was applied to the patient’s cognitive function by a physician who was unaware of the imaging findings. MoCA scale (Carlew et al., 2021) contains 7 cognitive domains: visuospatial and executive, naming, attention, language, abstraction, delayed recall and orientation, and is usually completed within 10 min. A MoCA score of < 26 was considered to be cognitive impairment.

### 3.4. Imaging

In this study, a 3.0T magnetic resonance scanner manufactured by GE, USA, was used for imaging the study subjects. All the patients in this study underwent conventional MRI (T1WI, T2WI, FLAIR, and DWI) and SWI of the skull, and all the imaging results were hidden from the patients’ basic information (The purpose of this is to avoid errors due to subjectivity and to ensure that the imaging results of each subject are evaluated without discrimination) and interpreted by two imaging physicians with more than 5 years of experience to determine the presence of CSVD imaging markers: CMBs, white matter hyperintensity (WMH), LI, enlarged perivascular space (EPVS). T1WI: repetition time (TR) = 450 ms, echo time (TE) = 14 ms; ② fast spin echo T2WI: TR 4 800 ms, TE 102 ms; ③ FLAIR: TR 8 600 ms, TE 102 ms; ④ DWI: TR 3,000 ms, TE 65 ms; ⑤ SWI: TR 3,000 ms, TE 65 ms; ⑤ SWI: TR 55 ms, TE 45 ms. The CMBs were graded according to the number of CMBs on the SWI of the head, and CMBs were grouped into 3 grades: 1∼2 for Grade 1, 3∼10 for Grade 2, >10 for Grade 3 (Lee et al., 2002). The locations of CMBs were classified into lobes, basal ganglia, thalamus, hippocampus, brainstem, cerebellum and mixed sites according to the microbleed anatomical rating scale (MARS) (Gregoire et al., 2009).

### 3.5. Detection of serum HMGB1 level

A total of 4 ml of early morning elbow venous blood was drawn from the enrolled patients in a non-anticoagulated blood collection tube, left at room temperature for 10 min, then centrifuged for 10 min using a centrifuge (3,000 r/min) to separate the upper layer of serum, and the serum HMGB1 level was measured by ELISA according to the kit instructions. In our study, we diluted the serum 10-fold, applied the HMGB1 kit for detection, and then expanded the data 10-fold to obtain the final results (Wuhan Fine Biotech Co., Ltd.).

### 3.6. Statistical treatment

Data were analyzed using SPSS 26.0 and GraphPad Prism 8.0. The measurement data that conformed to normal distribution were expressed as (*x* ± *s*), and two independent samples *t*-test was used for comparison between groups; the measurement data that did not conform to normal distribution were expressed as *M* (*P25*, *P75*), and Mann–Whitney *U* test was used for comparison between groups; the count data were expressed as relative numbers, and χ^2^ test was used for comparison between groups. Multivariable logistic regression analysis was performed to investigate the factors affecting cognitive impairment in patients with CMBs; *Pearson* correlation test was used to analyze the correlation between HMGB1 and different cognitive domains and the number of CMBs. ROC curves were used to evaluate the predictive value of HMGB1 on the occurrence of cognitive impairment in patients with CMBs. The differences were considered statistically significant at *P* < 0.05.

## 4. Results

### 4.1. Comparison of clinical data, biochemical data, and imaging markers between two groups of patients

Patients with cognitive impairment had significantly higher proportions of female, history of hypertension, occurrence of CMBs, number of CMBs >10, Lobes CMBs, and LI than those in the group without cognitive impairment (*P* < 0.05). Patients with cognitive impairment had significantly higher levels of HMGB1, UA, and HbAlc than the group without cognitive impairment (*P* < 0.05). The years of education of patients in the cognitive impairment group were significantly lower than those in the no cognitive impairment group (*P* < 0.05) (Tables 1–3).

**TABLE 1:** Comparison of clinical data between the two groups of patients.

Projects	Cognitive impairment group (*n* = 72)	No cognitive impairment group (*n* = 67)	*T/χ* ^2^	*P*
Age (years)	62.13 ± 8.96	60.78 ± 9.38	0.867	0.387
Education years (years)	6.72 ± 2.55	10.78 ± 3.05	8.506	<**0.001**
Gender (%)			3.985	**0.046**
Male	41 (56.9%)	49 (73.1%)		
Female	31 (43.1%)	18 (26.9%)		
**Past history (%)**				
Hypertension	57 (79.2%)	42 (62.7%)	12.109	<**0.001**
Diabetes	10 (13.9%)	11 (16.4%)	0.173	0.677
Heart disease	15 (20.8%)	17 (25.3%)	0.404	0.525
Cerebral infarction	44 (61.1%)	34 (50.7%)	1.514	0.219
Cerebral hemorrhage	6 (8.3%)	11 (16.4%)	2.113	0.146
Smoking	31 (43.1%)	30 (44.8%)	0.042	0.838
Drinking	20 (27.8%)	28 (41.8%)	3.015	0.083

The bold values represent the statistical difference.

**TABLE 2:** Comparison of biochemical data between the two groups of patients.

Projects	Cognitive impairment group (*n* = 72)	No cognitive impairment group (*n* = 67)	*Z*	*P*
HMGB1 (μg/L)	7.04 (5.89, 9.47)	4.00 (3.00, 5.56)	6.981	<**0.001**
UREA (mmol/L)	5.29 (4.44, 6.00)	5.26 (4.62, 5.71)	0.406	0.685
Cr (μmol/L)	65.10 (61.40, 69.43)	65.60 (58.50, 71.20)	0.091	0.927
UA (μmol/L)	282.50 (253.00, 340, 00)	261.00 (217.00, 287.00)	2.623	**0.010**
ALT (μmol/L)	18.00 (13.00, 36.25)	17.00 (13.00, 29.00)	1.679	0.095
AST (μmol/L)	18.00 (16.00, 24.75)	18.00 (15.00, 21.00)	1.868	0.064
TC (mmol/L)	4.02 (3.37, 4.99)	4.12 (3.16, 4.97)	0.022	0.983
TG (mmol/L)	1.22 (0.78, 1.57)	1.12 (0.85, 1.64)	0.433	0.666
LDL-C (mmol/L)	2.23 (1.85, 2.97)	2.31 (1.86, 2.71)	0.834	0.405
HDL-C (mmol/L)	1.11 (0.89, 1.41)	1.04 (0.91, 1.17)	1.262	0.209
FBG (mmol/L)	4.94 (4.43, 5.72)	5.09 (4.69, 5.59)	1.885	0.062
HbAlc (%)	5.60 (5.30, 5.97)	5.88 (5.31, 7.00)	2.765	**0.006**
Hcy (μmol/L)	17.36 (10.59, 28.25)	15.10 (10.90, 36.65)	0.783	0.435

The bold values represent the statistical difference.

**TABLE 3:** Comparison of imaging markers between the two groups of patients.

Projects	Cognitive impairment group (*n* = 72)	No cognitive impairment group (*n* = 67)	χ^2^	*P*
CMBs	38 (52.8%)	23 (34.3%)	4.797	**0.029**
Grade1: 1–2	4 (5.6%)	4 (6.0%)	0.011	0.916
Grade2: 3–10	14 (19.4%)	13 (19.4%)	0.000	0.995
Grade3: >10	20 (27.8%)	6 (9.0%)	8.086	**0.004**
Lobes	8 (11.1%)	2 (3.0%)	3.432	**0.050**
Basal ganglia	1 (1.4%)	4 (6.0%)	2.100	0.147
Thalamus	5 (6.9%)	5 (7.5%)	0.014	0.906
Hippocampus	3 (4.2%)	1 (1.5%)	0.888	0.346
brainstem	5 (6.9%)	3 (4.5%)	0.389	0.533
Cerebellum	1 (1.4%)	1 (1.5%)	0.003	0.959
Mixed areas	15 (20.8%)	7 (10.4%)	2.810	0.094
WMH	54 (75.0%)	47 (70.1%)	0.411	0.521
LI	46 (63.4%)	37 (55.2%)	4.302	**0.038**
EPVS	23 (31.9%)	24 (35.8%)	0.233	0.629

WMH, white matter hyperintensity; LI, lacunar infarction; EPVS, enlargement of perivascular space. The bold values represent the statistical difference.

### 4.2. Multivariable logistic regression analysis to investigate risk factors affecting cognitive function

In addition to the statistically significant indicators in Tables 1–3, including gender, years of education, history of hypertension, and levels of HMGB1, UA, and HbAlc, as well as the presence of CMBs and LI, the effects of age, history of cerebral infarction, history of cerebral hemorrhage, and other imaging markers of CSVD: WMH and EVPS, on cognitive function should also be considered. Therefore, in this study, Logistic regression analysis was performed with the presence or absence of cognitive impairment as the dependent variable and the above-mentioned indicators that may have an impact on cognitive function as independent variables. The results showed that HMGB1, UA, HbAlc, years of education and history of hypertension were strongly associated with the occurrence of cognitive impairment in patients with CSVD (*P* < 0.05) (Table 4).

**TABLE 4:** Multivariable logistic regression analysis to explore the risk factors for cognitive function.

Factor	β	*SE*	*Waldc* ^2^	*OR*	95% *CI*	*P*
Age	0.018	0.040	0.208	0.982	0.907–1.062	0.648
Education years	0.564	0.175	10.442	1.569	1.404–1.801	<**0.001**
Gender	0.495	0.710	0.486	1.640	0.408–6.592	0.486
Hypertension	1.998	0.799	6.259	1.136	1.028–1.649	**0.012**
Cerebral infarction	0.117	0.636	0.034	1.124	0.323–3.905	0.854
Cerebral hemorrhage	0.632	1.003	0.396	0.532	0.074–3.800	0.529
HMGB1	0.466	0.152	9.431	1.594	1.184–2.147	**0.002**
UA	0.010	0.004	7.604	1.010	1.003–1.018	**0.006**
HbAlc	0.627	0.296	4.503	1.534	1.299–1.953	**0.034**
CMBs	1.599	0.797	4.026	4.947	1.038–23.579	**0.045**
WMH	0.453	0.677	0.447	1.572	0.417–5.924	0.504
LI	1.457	0.694	4.407	1.233	1.060–1.908	**0.036**
EVPS	0.413	0.699	0.349	1.511	0.384–5.942	0.555

The bold values represent the statistical difference.

### 4.3. Comparison of cognitive abilities and correlation analysis between HMGB1 and various cognitive domains in the two groups

Patients with cognitive impairment had significantly lower MoCA total scores, visuospatial and executive abilities, and delayed recall abilities than those in the non-cognitively impaired group (*P* < 0.05). *Pearson* correlation analysis was used to explore the correlation between HMGB1 and each cognitive domain, and the results showed that HMGB1 levels were significantly negatively correlated with MoCA total score, visuospatial and executive abilities, and delayed recall abilities (*P* < 0.05) (Tables 5, 6).

**TABLE 5:** Comparison of various cognitive domains between the two groups of patients.

Projects	Cognitive impairment group (*n* = 72)	No cognitive impairment group (*n* = 67)	*T*	*P*
MoCA	13.83 ± 3.93	26.78 ± 1.05	6.051	<**0.001**
Visuospatial/executive	2.31 ± 1.20	2.95 ± 1.02	3.338	<**0.001**
Naming	2.38 ± 0.54	2.49 ± 0.76	0.924	0.357
Attention	5.51 ± 0.80	5.44 ± 0.68	0.521	0.603
Language	1.81 ± 0.65	1.77 ± 0.91	0.258	0.797
Abstraction	1.43 ± 0.62	1.56 ± 0.55	1.358	0.177
Delayed recall	1.55 ± 0.75	2.46 ± 1.48	3.876	<**0.001**
Orientation	5.48 ± 0.93	5.38 ± 0.83	0.651	0.516

The bold values represent the statistical difference.

**TABLE 6:** Correlation analysis between HMGB1 and each cognitive domain.

Projects	HMGB1	
	***r***	***P***
MoCA	−0.485	<**0.001**
Visuospatial/executive	−0.195	**0.022**
Naming	−0.142	0.096
Attention	−0.079	0.353
Language	−0.140	0.101
Abstraction	−0.147	0.085
Delayed recall	−0.374	<**0.001**
Orientation	−0.124	0.147

The bold values represent the statistical difference.

### 4.4. Univariate and multivariate Logistic regression analysis to explore risk factors for CMBs

The presence or absence of CMBs was used as the dependent variable, and age, gender, years of education, CSVD imaging markers, patients’ past history, and biochemical data were used as independent variables in univariate Logistic regression analysis, which showed that age, history of cerebral hemorrhage, history of heart disease, history of hypertension, history of smoking, history of drinking, HMGB1, UA, ALT, and AST were risk factors for CMBs (*P* < 0.05). Multivariate Logistic regression analysis was performed again with the above statistically significant indicators as independent variables, and the results showed that HMGB1, ALT, history of hypertension, and history of heart disease were strongly associated with the presence of CMBs (*P* < 0.05) (Table 7).

**TABLE 7:** Univariate and multivariate logistic regression analysis to explore the risk factors for CMBs.

	Univariate regression analysis *OR* (95% *CI*)	*P*	Multivariate regression analysis *OR* (95% *CI*)	*P*
Age	1.945 (1.908–1.984)	0.006	0.987 (0.931–1.047)	0.668
Gender	0.824 (0.407–1.668)	0.591		
**Imaging markers**				
WMH	0.712 (0.337–1.505)	0.374		
LI	1.072 (0.541–2.125)	0.841		
EPVS	1.050 (0.518–2.130)	0.892		
**Past history**				
Cerebral infarction	1.096 (0.557–2.154)	0.791		
Cerebral hemorrhage	3.576 (1.185–10.787)	0.024	4.123 (0.935–18.169)	0.061
Heart disease	2.500 (1.010–6.190)	0.048	5.502 (1.578–19.179)	**0.007**
Hypertension	12.214 (4.037–36.960)]	<0.001	14.208 (3.581–56.375)	<**0.001**
Diabetes	1.878 (0.734–4.800)	0.188		
Smoking	2.105 (1.062–4.170)	0.033	0.489 (0.070–3.412)	0.470
Drinking	2.814 (1.368–5.787)	0.005	3.447 (0.465–25.552)	0.226
**Biochemical data**				
HMGB1	1.163 (1.032–1.312)	0.014	1.414 (1.162–1.721)	<**0.001**
TC	0.899 (0.648–1.247)	0.524		
TG	0.929 (0.547–1.578)	0.785		
HDL-C	1.172 (1.051–1.587)	0.065		
LDL-C	0.904 (0.542–1.509)	0.700		
FBG	0.889 (0.720–1.098)	0.274		
HbA1c	0.924 (0.715–1.195)	0.547		
Hcy	1.015 (0.991–1.040)	0.220		
UREA	0.870 (0.664–1.140)	0.312		
Cr	0.989 (0.967–1.010)	0.302		
UA	1.005 (1.001–1.009)	0.024	1.003 (0.998–1.009)	0.251
ALT	1.066 (1.031–1.102)	0.000	1.076 (1.008–1.149)	**0.029**
AST	1.066 (1.010–1.125)	0.021	1.021 (0.916–1.138)	0.709

The bold values represent the statistical difference.

### 4.5. Correlation studies among HMGB1 levels, severity of CMBs, and cognitive impairment

**FIGURE 1:**
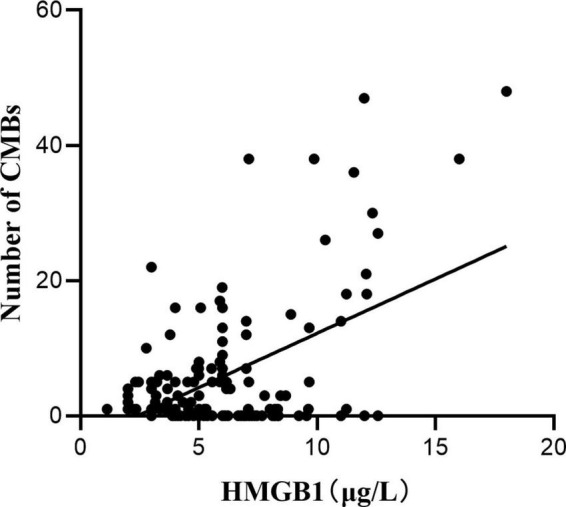
Correlation analysis between HMGB1 and the number of CMBs.

**FIGURE 2:**
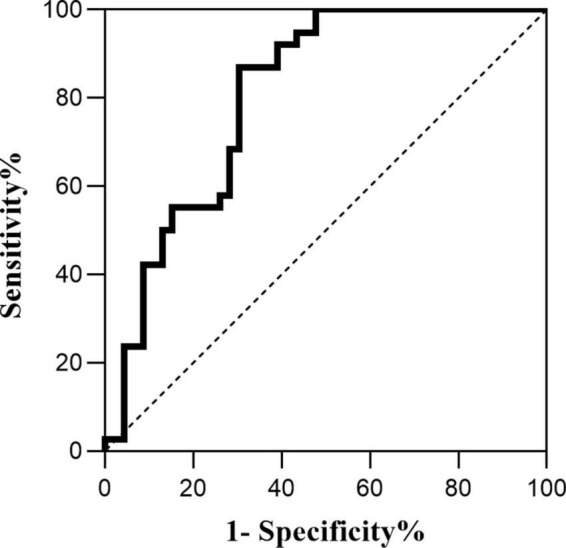
Predictive value of HMGB1 for cognitive impairment.

*Pearson* correlation analysis was used to explore the correlation between HMGB1 and CMBs, and the results showed a significant positive correlation between HMGB1 levels and the number of CMBs (*r* = 0.530, *P* < 0.05). ROC curve analysis of the predictive value of HMGB1 showed that HMGBI had a high predictive value for the development of cognitive impairment in patients with CMBs, and the area under the ROC curve was 0.807 (95% *CI*:0.683- 0.931, *P* < 0.001). The optimal threshold value was 5.04 μg/L, with a sensitivity of 86.8% and specificity of 69.6% (Figures 1, 2).

## 5. Discussion and conclusion

Cerebral microbleeds are focal deposits of blood degradation products contained in intracranial macrophages around blood vessels, and together with WMH, LI, and EPVS, they are known as imaging markers of CSVD (Peng et al., 2019). There is a correlation between the occurrence of CMBs and various physiological and pathological states. The most common symptom currently considered to be affected by CMBs is altered cognitive function (Meng et al., 2019). The consequences of their effects may depend on a variety of factors, including the number, size, and location of CMBs and the co-occurrence of other disorders. Research evidence suggests that greater numbers of CMBs are associated with more severe cognitive impairment and that the number of CMBs is an independent predictor of multiple cognitive domains and dementia severity (Miwa et al., 2014). A cross-sectional study has shown that CMBs are associated with impaired whole-brain cognition, reduced attention and processing speed, and impaired executive function (Yates et al., 2014). There is also an anatomical relationship between the distribution of CMBs and the degree of cognitive impairment and the cognitive domains impaired. A Meta-analysis showed that CMBs in the lobes, basal ganglia, and thalamus were associated with significant cognitive decline (Wu et al., 2014), and it has also been found that temporal CMBs are associated with memory and attention impairment, while frontal CMBs are associated with memory impairment, concept transfer, psychomotor speed and attention (van Norden et al., 2011). To date, however, the mechanisms by which CMBs contribute to cognitive impairment remain elusive. Initial studies suggested that CMBs may directly damage cortical, subcortical, and deep brain tissue through local deposition of iron-containing heme, leading to impairment of their connecting fiber tracts and neurotransmission pathways. With the development of neuroimmune science, many scholars have proposed that CMBs may lead to dysfunction of surrounding tissues, causing a sustained neuroinflammatory response with inflammatory mediators mediating oxidative stress damaging neurovascular units and causing local blood-brain barrier disruption (Eisele et al., 2016), microglia and perivascular macrophage activation, leading to cognitive impairment (Brundel et al., 2012; Del Brutto et al., 2016). The present study showed that CMBs were a risk factor for cognitive impairment, and the number of CMBs >10 and the location of CMBs in the lobes of the brain were significantly higher in the cognitively impaired group than in the non-cognitively impaired group, which is generally consistent with the results of previous studies. The present study also found that LI, history of hypertension, and low education level were also risk factors for cognitive impairment. LI caused neurological deficits and cognitive impairment by affecting cerebral blood supply. The number and volume of infarcts are important factors affecting cognitive performance; long-term hypertension causes impaired circulation in the deep white matter of the brain and changes in the BBB, leading to demyelination and affecting cognitive function; in patients with low education level, they are less stimulated by cultural knowledge, causing early decline of sensitive neurons, while high education level can resist age-related neuropsychological changes and slow down cognitive decline (Ding et al., 2019).

High Mobility Group Protein B1 is a highly conserved chromosomal binding protein, mainly found in the nucleus of eukaryotic cells, involved in gene replication, transcription and repair processes, and can also be actively secreted or passively released extracellularly to participate in the inflammatory response as an injury-associated molecule (Hisaoka-Nakashima et al., 2022). When monocytes-macrophages and astrocytes are exposed to exogenous pathogen-associated molecular patterns such as bacteria and viruses or endogenous inflammatory transmitters such as interleukin (IL)-1 and tumor necrosis factor-α (TNF-α), HMGB1 is actively secreted extracellularly. HMGB1 can bind to specific receptors and then promote the production of inflammatory factors through autocrine or paracrine forms to induce inflammatory responses; at the same time, HMGB1 itself has pro-inflammatory activity, which can directly cause BBB disruption and enhance inflammatory response (Manivannan et al., 2021). In addition to its pro-inflammatory activity, extracellular HMGBl also acts as a chemokine and growth factor, providing nutrition to cells, promoting neuroprotrusion and cell migration, and regulating the balance between autophagy and apoptosis. It has been shown that HMGBl is highly correlated with the development of cognitive impairment caused by acute brain injury, Parkinson’s, depression, epilepsy, and type 2 diabetes (Chu et al., 2013; Rolstad et al., 2015; Sun et al., 2015; Vidyanti et al., 2020; Piatkowska-Chmiel et al., 2021). However, the effect of HMGB1 on cognitive impairment in CSVD patients has rarely been investigated. In the present study, HMGB1 was found to be a risk factor for cognitive impairment in CSVD patients, and HMGB1 was significantly and negatively correlated with total MoCA score, visuospatial/executive abilities, and delayed recall abilities, the reason may be that the release of HMGB1 mediates the activation of microglia, and the oxidative stress and nitrative stress it causes aggravates neurovascular unit damage and BBB disruption, interfering with the electrophysiological activity of neurons and eventually causing degenerative necrosis of neurons, leading to cognitive impairment.

Previous findings have shown that abnormal UA and HbA1c also contribute to cognitive impairment. It is believed that UA is the end product of purine metabolism and that high UA levels affect cognitive function by promoting endothelial inflammatory responses and oxidative stress, leading to endothelial dysfunction and white matter damage (Ryu et al., 2013; Murthy et al., 2020); and in the hyperglycemic state, oxygen radicals are overexpressed, neurons experience impaired energy metabolism, and sustained anaerobic metabolism produces large amounts of lactic acid, causing damage to blood vessels and neurons (Bellary et al., 2021), which ultimately leads to cognitive impairment. The present study reached a consistent conclusion.

This study also identified HMGB1 as a risk factor for CMBs, and HMGB1 levels were significantly and positively correlated with the number of CMBs. The release of large amounts of HMGB1 may disrupt the tightly linked structure of the BBB, and the interaction between activated brain macrophages and astrocytes may further amplify the inflammatory response by producing chemokines that lead to BBB destruction by affecting endothelial cells or promoting the migration of monocytes (Dohgu et al., 2019). Several studies have shown that BBB disruption is involved in the pathological process of CMBs (Wang et al., 2021), triggering the formation of CMBs, and that the inflammatory response induced by CMBs disrupts endothelial cell function, exacerbating BBB disruption and further increasing the permeability of the BBB. The increase in the number of CMBs, the increase in the expression level of HMGB1, and the increase in BBB disruption suggest that neuroinflammation has a cumulative effect on the disruption of CMBs and the BBB. In this study, ROC curve analysis of HMGB1 expression levels in patients with CMBs showed that HMGBI has a high predictive value for the development of cognitive impairment in patients with CMBs, implying that HMGB1 may produce neuroinflammatory effects through sustained release, destroying neurovascular units and the BBB, which results in the continuous generation of CMBs in various location of the brain and ultimately leads to cognitive impairment.

In summary, this study concluded that there is a close association between HMGB1, CMBs, BBB and cognitive impairment, and any change in any of these indicators may have a significant impact on the course of the disease. HMGB1 is involved in the pathogenesis of many inflammatory and autoimmune diseases as an important extracellular damage molecule. Therefore, HMGBl may serve as a new therapeutic target for patients with CMBs, providing a novel direction for the prevention and treatment of cognitive impairment, which has important implications for the early detection, treatment efficacy and prognostic assessment of patients with cognitive impairment.

## 6. Limitations and prospects

The present study is only a single-center, cross-sectional study and has a relatively small sample size. It also lacks theoretical results from animal experiments to provide more powerful support. In the future, we aim to conduct a multicenter, large sample size investigative study, as well as produce animal models of CMBs to explore the specific pathway mechanisms by which HMGB1 exerts its neuroinflammatory effects, in order to provide clinicians with help in early intervention and control of the occurrence and development of neuroinflammation.

## Data availability statement

The raw data supporting the conclusions of this article will be made available by the authors, without undue reservation.

## Ethics statement

The studies involving human participants were reviewed and approved by the Ethics Committee of the First Affiliated Hospital of Xinxiang Medical University. The patients/participants provided their written informed consent to participate in this study.

## Author contributions

MW and JZ: conceptualization and formal analysis. MW, JZ, and SL: methodology. JL and FW: software. JZ: validation, resources, and writing—review and editing. MW and FW: investigation. MW: writing—original draft. QL and SJ: visualization. JZ and SJ: supervision. SL and CL: project administration. All authors have read and agreed to the published version of the manuscript.
